# Recovery of visual fields in brain-lesioned patients by reaction perimetry treatment

**DOI:** 10.1186/1743-0003-4-31

**Published:** 2007-08-16

**Authors:** Fritz Schmielau, Edward K Wong

**Affiliations:** 1 Institute for Medical Psychology and Special Neurorehabilitation, University of Lübeck, Germany; 2 Department of Ophthalmology, University of California Irvine, USA

## Abstract

**Background:**

The efficacy of treatment in hemianopic patients to restore missing vision is controversial. So far, successful techniques require laborious stimulus presentation or restrict improvements to selected visual field areas. Due to the large number of brain-damaged patients suffering from visual field defects, there is a need for an efficient automated treatment of the total visual field.

**Methods:**

A customized treatment was developed for the reaction perimeter, permitting a time-saving adaptive-stimulus presentation under conditions of maximum attention. Twenty hemianopic patients, without visual neglect, were treated twice weekly for an average of 8.2 months starting 24.2 months after the insult. Each treatment session averaged 45 min in duration.

**Results:**

In 17 out of 20 patients a significant and stable increase of the visual field size (average 11.3° ± 8.1) was observed as well as improvement of the detection rate in the defective visual field (average 18.6% ± 13.5). A two-factor cluster analysis demonstrated that binocular treatment was in general more effective in augmenting the visual detection rate than monocular. Four out of five patients with a visual field increase larger than 10° suffered from hemorrhage, whereas all seven patients with an increase of 5° or less suffered from infarction. Most patients reported that visual field restoration correlated with improvement of visual-related activities of daily living.

**Conclusion:**

Rehabilitation treatment with the Lubeck Reaction Perimeter is a new and efficient method to restore part of the visual field in hemianopia. Since successful transfer of treatment effects to the occluded eye is achieved under monocular treatment conditions, it is hypothesized that the damaged visual cortex itself is the structure in which recovery takes place.

## Background

There are only a few known treatment approaches to restore loss of vision due to a cerebrovascular accident (CVA or stroke) of the posterior part of the brain. Impairment of visual function, among which corresponding visual field loss in both eyes (homonymous hemianopia) is the most common type, will result in legal blindness so that one has difficulties to read, orientate oneself, ambulate or drive a vehicle.

In some of these patients, there may be spontaneous recovery of vision loss, usually within the first weeks or months after the incident [1].

After early recovery in the first few months, few studies describe attempts to treat homonymous hemianopia. Zihl and von Cramon [2,3] report that the repetitive presentation of threshold stimuli in the transition zone between the intact and defective visual field (VF), or the saccadic localization of targets presented within the anopic field may result in increased VF up to 27° (a) and 48° (b) of visual angle, respectively. In a replication study, however, Balliet et al. [4] observed an average increase of less than 1 degree as a consequence of the same treatment.

Recent evidence in favor of treatment effects, derives from an attempt to reduce VF defects in patients with post-chiasmatic and optic nerve injuries by using a personal computer monitor for stimulation [5]. The authors claim that sequential suprathreshold stimulus presentations in 150 training sessions within the defective VF resulted in an average increase of detection rate of 29% in post-chiasmatic and 74% in optic nerve patients, when diagnosed with static perimetry. According to conventional static perimetry testing used as a secondary outcome measure, however, the group of post-chiasmatic patients did not show any training effect (0.43° ± 0.34). Support for the recovery hypothesis is also given by earlier studies in primates, demonstrating that after discrete striate cortex ablations, a decrease of the scotoma size was obtained by visual discrimination testing as a training method or by saccadic eye movement training [6-9].

## Methods

**Figure 1:**
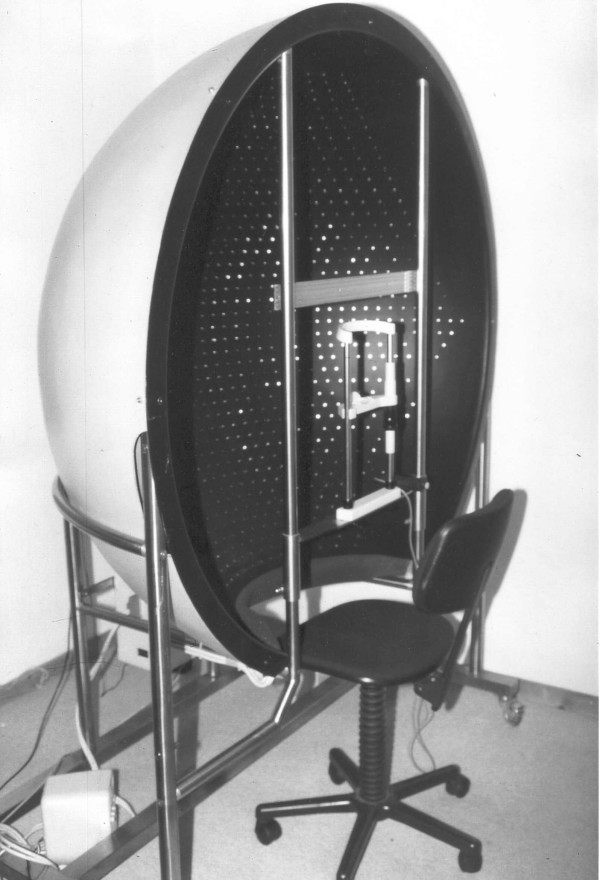
Lubeck Reaction Perimeter.

To evaluate whether restoration of VF in patients with homonymous hemianopia is possible, and if so, to improve the efficacy of treatment, the Lubeck Reaction Perimeter (LRP) (fig. 1) was designed [10,11].

### The Lubeck Reaction Perimeter

The basic construction of the perimeter is a hemisphere with an inner radius of 70 cm. This size has been selected to guarantee a minimum of accommodation strain for the patient. Background luminance of the inner hemisphere surface is equal to 0.014 cd/m^2^. At the pole of the hemisphere, a red LED serves as a fixation element. 1740 green LEDs (∅ = 30', dominant wavelength λ = 571 nm, maximum luminance L = 3200 cd/m^2^) are used as light stimulus elements. They are distributed homogeneously at a distance of 3° within the inner hemisphere on isoazimuth- and isoelevation-lines. Luminance values of LEDs may be modified in steps of 0.2 logarithmic units. Two loudspeakers for auditory stimulation are situated below the hemisphere. A personal computer uses a special software permitting a) assessment of the VF size by random presentation of stimuli, and b) treatment by sequential and repetitive stimulation of LEDs in order to focus the patient's selective attention to stimulated areas. Patients must respond to the detection of any lit LED by pressing a reaction-time key. Each visual stimulus is announced by a random (500 – 2000 ms) auditory warning stimulus (f = 1000 Hz) to raise attention before visual stimulation. Reaction times have to fall into a time window of 150 – 900 ms after the visual stimulus. In the assessment mode a variety of fixed LED arrays with different stimulus densities and distributions can be tested. These software options allow for fast and comprehensive surveys of the size of the VFs and assessment times between 5 and 45 minutes, including automated breaks every 3 – 5 minutes to prevent fatigue. All locations and reaction times are stored; median, arithmetic mean, standard deviation and type and number of errors are calculated. The reaction time distribution within the VF is presented in different colors on a PC monitor and may be printed.

### Treatment

**Figure 2:**
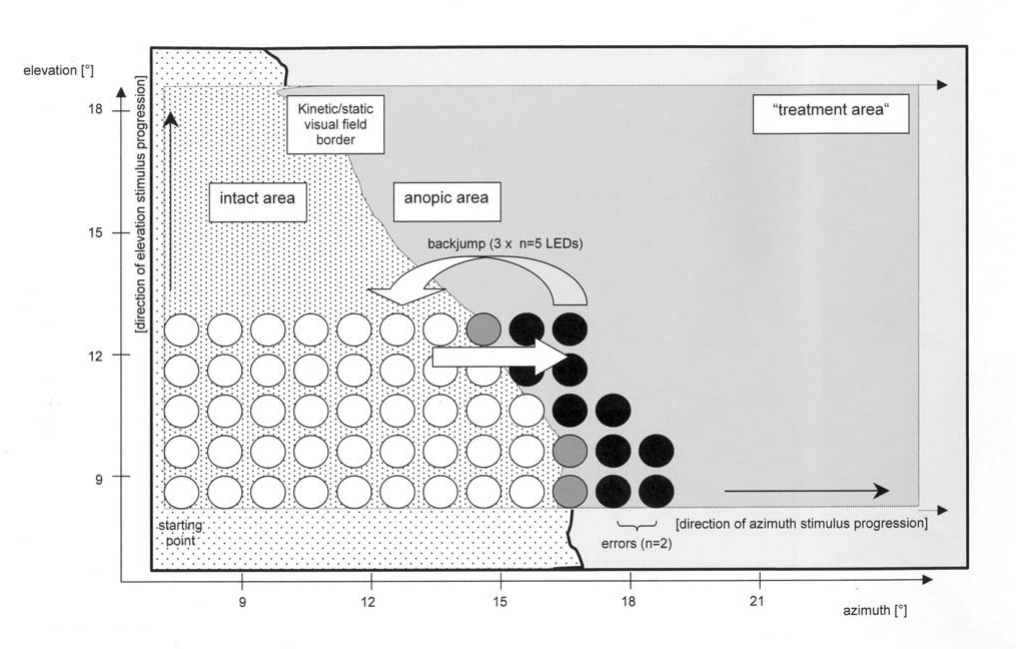
**Treatment algorithm**. Section of the visual field of a patient including an area of intact (dotted) and anopic (light shading) vision. The "treatment area" (dark shading) is a rectangular area in which LEDs are stimulated in a systematic way (here from left to right). White spots: normal reaction times. Grey spots: prolonged reaction times within the reaction interval. Black spots: delayed or no reactions (errors). See text for details.

Since most of the studies on brain plasticity research assume that selective attention plays a key role for VF recovery treatment, special efforts were undertaken to ensure a high level of attention whenever visual stimuli were presented. To perform a most active role during treatment, the patient must respond immediately by pushing a button ("simple-reaction-time paradigm") whenever a light stimulus was perceived. Simple reaction times (SRTs) measured the performance level. There is a close relationship between SRT prolongation and threshold augmentation, a characteristic feature of defective visual fields [12]. While fixating on the central LED, 100 ms flashes were shown in a pre-selected area of the patient's VF (i.e. "treatment area"). Fixation was controlled by monitoring eye movements with a low luminance sensitive video camera, sessions with improper fixation being rejected. Measurements demonstrated an accuracy to detect eye shifts of 1° amplitude. The treatment area always included areas of intact VF. Stimulation started within the intact VF and successively moved into the anopic VF area. Chance responses were very unlikely due to the small time window of allowed SRTs after the random auditory warning signal. In the case of no or delayed (901 – 1400 ms) response, a low frequency (f = 500 Hz) tone was given as a negative feedback signal to increase the patient's attention. When the patient failed to respond to two successive stimuli, the next stimulus began 12° back, where perception had been successful. This procedure was repeated three times before stimulating on the next iso-elevation or iso-azimuth line (fig. 2).

The advantage of this adaptive treatment algorithm is that stimulation is automatically adjusted to the current VF border and is concentrated on the transition zone between the intact and defective VF. Within a typical 45 minutes treatment session, about 500 stimuli were presented in the treatment area.

### Measurement of visual field size

To calculate improvement in kinetic perimetry (Goldmann-type Tubingen perimeter) due to treatment, the difference of the damaged kinetic VF before and after treatment was calculated. Each time the VF size was obtained by measuring the VF extension along each 15° meridian including the 90° and 270°meridian (vertical) and calculating the average. The change of detection rate (static perimetry) within the damaged VF due to treatment, as measured with the LRP was obtained by calculating the difference before and after treatment. At any time the detection rate was calculated as the percentage of correct responses within the whole hemifield. Within an intact hemifield 350 responses were possible. If for example a patient gave 100 correct responses before his treatment, his detection rate at that time would be 100:350 = 28.6%. If for example he performed 130 correct responses when tested after treatment, his detection rate would be 130:350 = 37.1%. The improvement due to treatment in that case would be 37.1% - 28.6% = 8.5%.

### Patients

Patients were included in the study who met the following criteria: no ocular or oculomotor pathologies, no fixation instabilities, a corrected visual acuity of ≥ 0.67, no permanent attentional deficits (neglect), no major motor deficits, no pronounced memory, speech or intellectual deficits. Line bisection tests and behavioural testing were performed to exclude neglect. Investigational approval was given by the University of Luebeck Medical Ethics Committee. Twenty right-handed patients with homonymous hemianopic visual defects resulting from cerebral lesions were selected on the basis of regular patient availability and motivation to participate in a long-time study of approximately one year. Lesions included infarction (n = 11), hemorrhage (n = 7) and closed head trauma with post-traumatic subdural hematomas (n = 2). Lesion sites and size were documented by computed tomography (CT) or magnetic resonance imaging (MRI). Before treatment, all patients received careful neurological and ophthalmological evaluation. The average age was 53.5 years (range: 21 – 80). Nine patients were female and eleven were male. Nine patients suffered from additional paresis mostly of the brachio-facial type which, however, permitted them to climb the stairs to the third-floor of our institute building. Before and after perimetry treatment, vital eye parameters (intraocular pressure, fundus and optic nerve papilla) and basic visual functions such as acuity, binocular fusion, stereopsis, central und peripheral form and color vision, incremental thresholds, critical flicker fusion frequency (CFF), were measured. In some selected patients only brightness perception, visual evoked potentials (VEP) and the patient's ability to localize visual and auditory stimuli in space were investigated. Assessment of vital eye parameters and visual subfunctions was performed at least at base line and at the end of treatment in most patients, however; the assessment was performed after several post-treatment intervals (ranging from six months to more than ten years).

VF evaluations were performed with manual-kinetic perimetry with the Tubingen Perimeter and automatic static perimetry with the LRP. Patients were asked to perform "exercise" perimetry measurements to familiarize themselves with the measuring procedures and to obtain stable pre-training VFs and to establish a stable perceptual criterion when a stimulus is evaluated as seen and must be responded to. Both types of measurements were done at least three times: at baseline, at the end of treatment and after an interval of six months up to more than ten years. Besides measuring the treatment outcome and stability of treatment effects ourselves by using two types of perimetry (automatic static and kinetic) and other functions, in some of the out-of-town patients conventional threshold perimetry was performed by the patient's ophthalmologists, not involved in this study.

**Figure 3:**
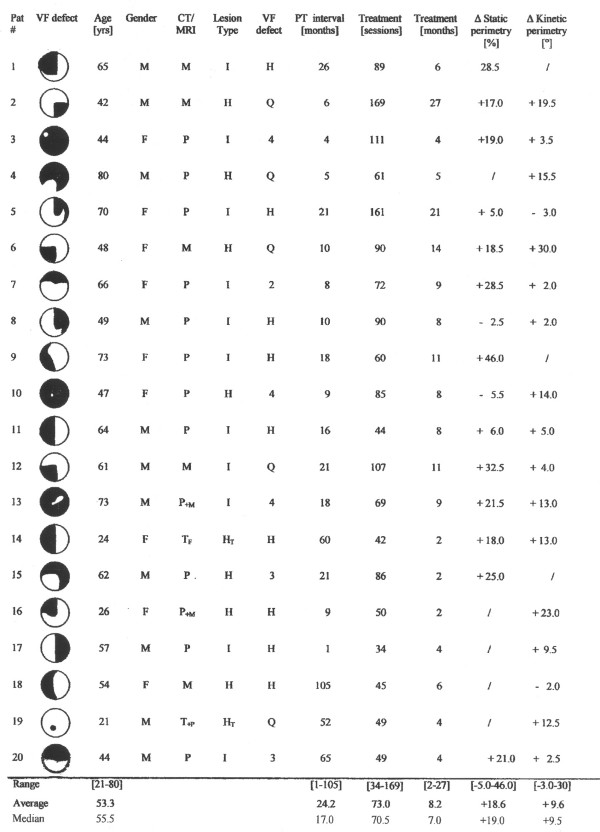
**Clinical description and treatment results**. **CT/MRI**: lesion site M = medial cerebral artery P = posterior cerebral artery P_+M _= posterior and medial cerebral artery T = trauma including frontal _F _or posterior _P _lesion. **lesion type**: I = infarction H = hemorrhage H_T _= trauma including intracerebral hemorrhage **VF defect**: Q = quadrantanopia H = hemianopia 2 = upper two quadrants 3, 4 three or four quadrants **PT interval **= duration of post traumatic interval **Δ static perimetry **= change of detection rate in defective half-field after treatment **Δ kinetic perimetry **= change of VF size in defective half-field after treatment. The left column "VF defect" represents a schematic drawing. VF defects were similar for both eyes.

The interval between lesion and onset of treatment ranged from 1 to 105 months (average: 24.2 ± 26.4 months); in only three patients (# 3, 4, 17) it was shorter than six months. In eight patients no spontaneous VF recovery had been observed; in twelve patients spontaneous improvements of VF size had occurred before treatment. See fig. 3.

Within an average time of 8.2 months (range: 2 – 27) a mean number of 73.0 ± 31.8 (range: 34 – 169) treatment sessions (approximately two per week) were performed by each patient. Treatment was executed with both eyes open in 13 patients, while 7 randomly selected patients were treated with one eye patched to test for interocular transfer of treatment effects to evaluate the cortical effects of treatment. The majority (n = 13) of patients were treated binocularly since that type of treatment was reported to be less exhausting with respect to maintaining fixation and keeping attention than monocular training.

To keep the patient's motivation at a high level and complete therapy, each patient was informed about his respective daily treatment performance at the end of each treatment session. In addition, patients were asked from time to time, or reported themselves spontaneously, whether they experienced treatment-related improvements of visual functions, such as VF size, acuity and behavior, e.g. reading or bumping into obstacles/activities of daily living (ADL).

No control group was included within the study since 1) in the majority of patients the stability of pre-treatment VF size was guaranteed by comparing the results of external perimetry (usually several years old) with our own pre-treatment results 2) control of spontaneous recovery in patients with fresh lesions (< six months; n = 3) was critically investigated by treating only one part of the defective field at a time and comparing the changes in the treated and non-treated VF part (within-patient control). Of the patients (#3, 4, 17) with fresh lesions, no VF improvement was observed in the non-treated VF area. 4) motivation has proved to be a very important parameter in the patient's will to participate in a long-term study, coming several times a week over a nine-month period to our institute, to perform a very demanding and sometimes frustrating treatment. Beyond ethical objections against a sham treatment, it would have been very unlikely that our patients could have been permanently motivated to participate in such a long-time therapy study from which the did not perceive any improvements over the months, even if a visual handicap such as hemianopia represents a serious motivation to perform therapy

## Results

**Figure 4:**
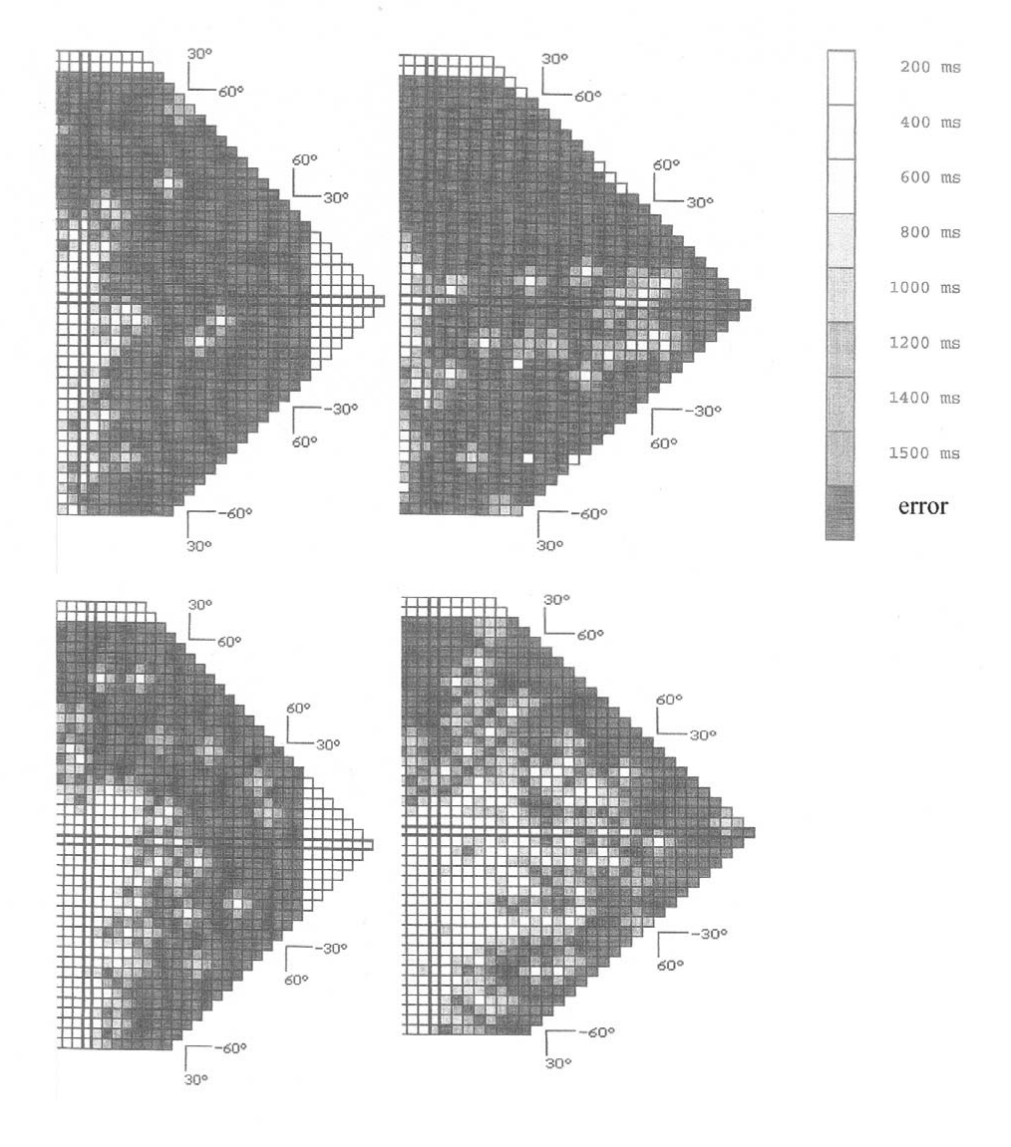
**Increase of visual fields due to treatment**. Visual fields of a 62 years old patient (#15) before and after treatment. upper: before treatment. lower: after treatment. left: left eye. right: right eye. The defective right visual hemifield and a central part of the left hemifield are shown. Hemianopia was caused by a bilateral lesion of the posterior cerebral artery 21 months before the onset of treatment. 86 treatment sessions within two months increased the detection rate in the right hemifield by 25%.

The outcome of sensori-motor treatment, using the self-adaptive stimulus algorithm of the LRP, was measured with manual-kinetic and/or automated static perimetry. Due to time restrictions before treatment, in twelve patients only both types of perimetry testing could be performed. In the majority of patients, treatment had been effective, resulting in a distinct increase of their VF size and stimulus detection rate within the former anopic VF area. An example of treatment efficacy in a patient suffering from a bilateral occipital lesion and a consecutive VF defect in both hemifields is given in fig. 4.

### Kinetic perimetry outcome

**Figure 5:**
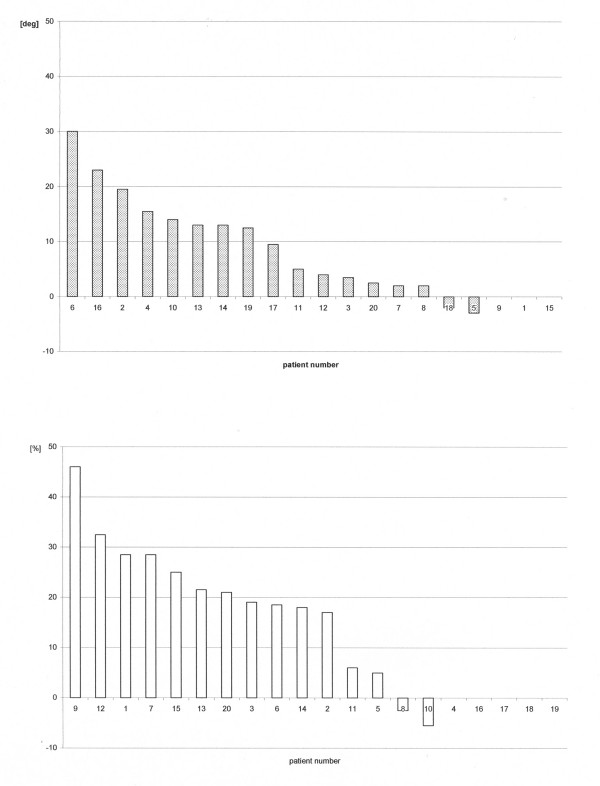
**Ranking of changes due to treatment**. Upper graph: changes in visual field size (kinetic perimetry). Lower graph: changes in detection rate (static perimetry)

In 17 of 20 treated patients, VF size was assessed by manual-kinetic perimetry. Of these 17 patients, 15 demonstrated an average VF increase of 11.3° ± 8.1 (range 2 to +30°) for both eyes averaged (fig. 3). There were only two patients of 17 who demonstrated a slight reduction (-2°, – 3°) of their kinetically measured VF after treatment. In one of them (#5), however, the detection rate in static perimetry in his damaged right VF after treatment had increased by 5%. The other patient (#18) was the only patient who had permanent fixation difficulties during treatment. Suffering from a right hemisphere hemorrhage of the middle cerebral artery, she never maintained precise fixation when stimuli were presented in her treatment area though she was able to fixate properly during randomized VF assessment. Including those two patients with minor VF losses, the average increase of the whole group of 17 patients due to treatment was 9.6° ± 8.8 (fig. 3). Out of the 15 patients demonstrating VF increase, the VF enlargement ranged a) in seven patients below 10° b) in six patients between 11 and 20°, and c) in two patients between 21 and 30° (fig. 5 upper part).

### Static perimetry outcome

Static perimetry was used in 15 out of 20 patients to evaluate the outcome of LRP treatment. Due to time constraints before treatment, in the remaining five VF size before and after treatment was measured only by kinetic perimetry. The average improvement of detection rate for both eyes within the defective VF was 18.6 % ± 13.5 (range: -5.5 to + 46 %). Since the average detection rate is based on a normal VF size which is given a detection rate of 100%, an increase of the detection rate of 18.6 % indicates an increase of usable VF area by 18.6 %, nearly one-fifth of a normal VF. In only two patients the detection rate decreased during treatment by -2.5% and by -5.5 % (# 8, 10). In both patients, however, kinetic perimetry demonstrated a slight (#8: +2°), or a moderate (#10: +14°) increase of VF size. Out of the 13 patients demonstrating an increase of detection rate, the average binocular increase ranged a) below 10% in two patients, b) between 10 and 20% in four patients, and c) between 20 and 46% in seven patients (fig. 5 lower part).

### Stato-kinetic dissociation

When comparing changes in static and kinetic perimetry due to VF treatment in individual patients who had been investigated with both types of perimetry (n = 12), three patient clusters can be distinguished: patients who showed similar changes in both types of perimetry (cluster A consisting of two patients # 2, 11), patients in whom changes in static perimetry were larger than in kinetic perimetry (cluster B) containing seven patients #3, 5, 7, 12 – 14, 20, and patients in whom changes in static perimetry were smaller than in kinetic perimetry (cluster C) three patients # 6, 8, 10).

In cluster A, changes in static perimetry average 11.5% (range +6 to +17%), changes in kinetic perimetry average 12.25° (range +5 to +19,5°). In cluster B average change in static perimetry equals 20.8% (range +5 to +32.5%) whereas the average change in kinetic perimetry is much smaller: 5° (range -3.0 to +13.0°). In cluster C the average change in static perimetry equals 3.5% (range -5.5% to +18.5%), whereas the average change in kinetic perimetry equals 15.3° (range +2 to 30°). No significant correlation, however, was found between the degree of change demonstrated by static and by dynamic perimetry when the data of all patients were pooled into one group.

### Stability of VF increase after the end of treatment

In 15 of 20 patients, VF size (kinetic perimetry) and detection rate (static perimetry) were investigated at least six months after the end of treatment; 13 demonstrated no decrease of VF size and detection rate. In one infarction patient (# 5) who had demonstrated an ambiguous treatment outcome, detection rate had further increased (+4%) during the follow-up interval of twelve months, whereas the kinetically measured VF size had again decreased by 2° after a 3° reduction during treatment. In another patient (#6) within one year after the treatment, a second hemorrhage of her right middle cerebral artery had occurred which completely reversed her treatment-induced VF improvement of 30°. It finally resulted in a nearly complete hemianopia of her left VF, whereas the result of the first insult had been only a quadrantanopia. Several months of treating that second defect, however, was much less effective than treating the initial one.

**Figure 6:**
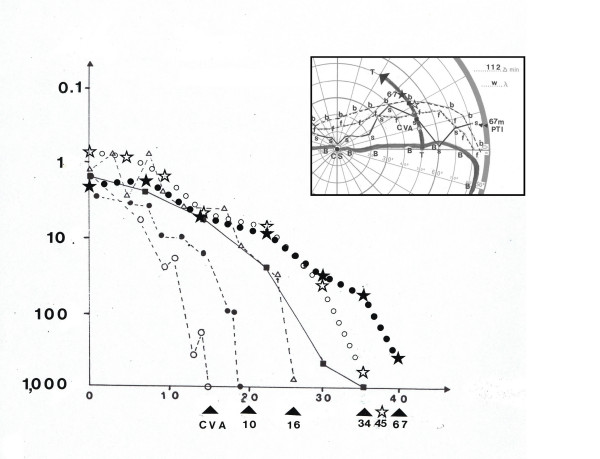
**Change of threshold curves within a 67 months post traumatic interval**. Large image. Incremental thresholds along a circle of 50 deg eccentricity (zenith angle Φ) of the treated upper right VF quadrant in a patient (# 20) suffering from a bilateral infarction of the posterior cerebral arteries and a simultaneous infarction of the left thalamus at different times after CVA. **CVA **1 month after bilateral insult, immediately before the beginning of treatment with LRP. **10, 16, 34, 45, 67 **refer to measurements at post traumatic interval (**PTI) **months after **CVA**. **Open circles **= 1 month after CVA **Filled circles **= **CVA **+ 10 months at the end of treatment phase 1 (= 11 months after CVA) **Open triangles **= **CVA **+ 16 months **Filled squares **= **CVA **+ 34 months **Open stars **= **CVA **+ 45 months **Filled stars **= **CVA **+ 67 months. **Abscissa **= polar (or azimuth angle) Θ [deg] **0 **= right horizontal meridian **90 **= upper vertical meridian **Ordinate **= incremental (luminance) threshold T = ΔL/L, background luminance L = 3,2 cd/m^2^, stimulus duration Δ t = 200 ms, circular stimulus ∅; = 112', stimulus color = white. Small image. Subjective brightness estimation, and VF borders of threshold, brightness and form perception within a part of the central VF of pat #20 with accentuation of the treated (phase 1) upper right VF quadrant. **CS **central comparison stimulus (subjective brightness : 100) **CVA **VF border on eccentricity circle Φ = 50° when measured by incremental thresholds T one week after CVA **symbols **VF border (incremental threshold T) as in large image **67 **VF border (incremental threshold T) 67 months after CVA **67 months PTI (**and curve of **filled squares) **subjective brightness estimation curve "100 %" (= to brightness of CS) 67 months after CVA **b **VF border (brightness perception) measured by kinetic perimetry 67 months after CVA **f **VF border for form perception (100% correct discrimination of white circles and squares of equal size and luminance measured by kinetic perimetry.

In one patient (#20) suffering from a bilateral lesion of the posterior cerebral artery, stability of treatment effects have been demonstrated for more than ten years. Fig. 6 shows the results of the first 5 years and 7 months after CVA for a selected area and a zenith angle of Θ = 50°. When first measured by kinetic perimetry, the VF border (for the detection of white light) in the upper right VF quadrant ran close to the horizontal meridian (Θ = 0°), in the left VF even far below the horizontal (Θ = 180°) meridian within the lower left VF quadrant. After some spontaneous recovery of the whole VF, treatment of the upper right quadrant (phase I = 89 sessions, starting one month after CVA) resulted in an increase of only the treated quadrant. The course of incremental threshold T curves for Θ = 50° over the whole period of 67 months (fig.6 large image) demonstrates a VF border shift ΔΦ of approx. four degrees and a general decrease of T within 9 months (fig. 6 large image from **CVA **to **10**). Within another 66 months, the VF border was step-by-step shifted into the anopic quadrant (towards Φ = 40° at **67**) and T was gradually lowered. In parallel to that VF border shift and decrease of the incremental threshold, the magnitude of perceived subjective brightness of test stimuli – when comparing to a foveal comparison stimulus CS of equal size and luminance – within the restituted VF increased. After a post traumatic interval PTI of total 67 months, as demonstrated in fig. 6 small image, the course of the 100% subjective brightness curve in the upper right quadrant (**67 months PTI**) was located far in the former anopic VF area. Its location increased with increasing eccentricity (= zenith angle Θ) demonstrating a maximum at Θ = 40°. With increasing Θ the difference between the VF border for detection of light (fig. 6 small image **b**) and perceived 100% brightness (**s**) first increases (0 ≤ Θ ≤ 20°) then decreases gradually again towards 2° at Φ = 40°. At this position (Φ = 40°), close to the VF border, patient #20 thus perceived a stimulus as bright (= 100%) as at the foveal VF position. At that time, seventy-six months after CVA, the VF border for the detection of form (curve **f **in fig. 6 small image) ran far in the former anopic VF at a distance ΔΦ of about 5 to 10° within the VF for brightness detection (curve **b **in fig. 6). And visual acuity had recovered from 0,1 to 0,7.

**Figure 7:**
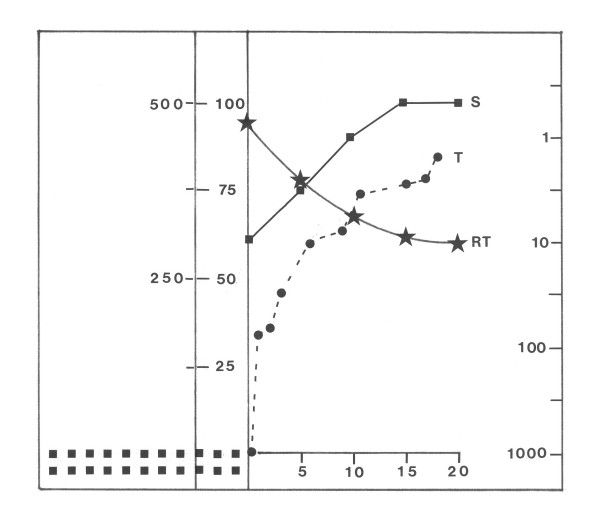
**Change of different visual parameters from intact to anopic VF after LRP treatment in the upper right VF quadrant**. Reaction times **RT**, incremental thresholds **T **and perceived (subjective) brightness **S **as a function of proximity to the anopic VF area. Same patient (# 20) as in fig. 6. Data were collected at post traumatic interval **PTI 10 **10 months after **CVA **(after the end of treatment phase I); note that (fig. 6) = 11 months after CVA. **Ordinate: stars = **RT [ms], **squares = **magnitude of subjective brightness S [%], **circles = **incremental threshold T;**Abscissa: **distance from kinetic VF border ΔΦ [°]. See text for details.

In fig. 7 the course of reaction times RT, thresholds T and (subjective) perceived brightness estimations S of patient # 20 after treatment (**10 months **after **CVA **as in fig. 5 = 11 months after insult) are shown for an eccentricity = zenith angle of θ = 50° as a function of the distance (ΔΦ) from the anopic VF border, to especially demonstrate the close relationship between particular visual parameters. As a consequence of treatment, at θ = 50° the VF border (kinetic perimetry) had between displaced into the anopic area by ΔΦ = 8° and T had been lowered between within an area of 15° width [3 < Φ ≤ 18°] up to a factor of 16. As demonstrated in fig. 6, T increases from intact to anopic VF, from approx. T = 1 at the horizontal meridian (Φ = 0°, Θ = 50°) to T = 1,000 at Φ = 18 °, Θ = 50° (VF border as determined by measurements of T, detection rate > 50%). As can been seen from fig. 7, with approaching the anopic VF from ΔΦ = 15° to 0°, RT increases exponentially: from 320 ms to 470 ms, whereas the magnitude of perceived brightness S decreases from 100% to 61%. Simultaneously the quality and size of the perceived stimuli change from clear to diffuse ("like the moon behind clouds") and small to large, when a stimulus is presented at ΔΦ = 0. The function RT = f (S) can be a approximated by a linear function (r = -0.8): with increasing brightness by 25%, reaction time decreases by approx. 40 ms.

This example may demonstrate that more than four years after the end of the initial treatment (phase I), the VF size for brightness and form detection, as measured with kinetic perimetry, had increased considerably, compared to the end of spontaneous recovery and beginning of the treatment, one month after CVA. Within the restituted VF, the quality of vision, as indicated by low thresholds, good form and brightness perception was stable for another seven years (total follow up interval was 13 years).

### Group differences of treatment outcome

#### Monocular versus binocular treatment

In seven randomly selected patients treatment was performed with only one eye open to test for transfer of treatment effects to the occluded eye. In six of those seven patients (# 5, 7, 8, 10, 11, 16, 19) treated monocularly, treatment resulted in VF enlargement (kinetic perimetry) in the treated as well as in the non-treated eye. In only one patient kinetic perimetry did not reveal a positive treatment effect (# 5). For the whole group of patients treated monocularly, the occluded eye showed about the same improvement from treatment as the open eye did: a +6.2% ± 16.4 increase of detection rate and 8.9° ± 11.9 increase of VF size in the occluded and a +6.6% ± 12.5 increase in detection rate as well as a 7.8° ± 6.8 increase of VF size in the open eye.

A two factor cluster analysis was performed to test for the influence of the type of treatment ocularity (monocular versus binocular; factor 1) and of the type of the lesion (infarction versus hemorrhage; factor 2) on the LRP treatment outcome. When static perimetry was used as an outcome measure cluster analysis demonstrated three clusters. The average change of detection rate equals 19.625 % ± 3.64 (cluster 1), 28.08 % ± 10.17 (cluster 2) and 6.30 % ± 13.34 (cluster 3). Within the two clusters demonstrating the highest increase of detection rate due to treatment there were only patients (n = 6 in cluster 2 and n = 4 in cluster 1) who had performed the treatment binocularly, whereas in cluster 3 with the lowest increase there were only patients (n = 5) who had performed the treatment monocularly. Though monocular treatment is apparently effective in raising the detection rate, binocular treatment has three to four times the effect of monocular treatment. Out of the eight patients of fig. 5 upper part demonstrating an increase of detection rate above 10%, seven had performed the treatment with both eyes open, whereas the four patients with the smallest improvements were treated monocularly.

When the above two factor cluster analysis was applied to change of VF size in degrees of visual angle obtained by kinetic perimetry, results were less obvious. In this case 2 clusters were obtained. Cluster 1 demonstrating an average VF size increase of 15.69° ± 9.30 contains three monocularly and five binocularly treated patients whereas cluster 2 showing an average VF size increase of 4.28° ± 4.62 includes 4 monocularly and five binocularly treated patients.

#### Nature of lesion

As mentioned in section 4.1, a two factor cluster analysis with the factors 1) ocularity of treatment and 2) type of the lesion had been performed to search for factors influencing the degree of treatment outcome in different patients. In eleven patients VF defects had been caused by infarction, in nine patients by hemorrhage (n = 7) or closed brain trauma with post-traumatic subdural hematomas (n = 2). Within the cluster analysis treatment efficacy was analyzed with respect to the nature of the lesion (factor 2), comparing treatment outcome in patients with infarction and hemorrhage. The group of patients suffering from hemorrhage included both patients with subdural hematomas.

When change of the VF size in kinetic perimetry was used as a treatment outcome variable, the cluster analysis demonstrated two clusters differing significantly in the degree of treatment outcome. In cluster 1 the average VF enlargement equals 15.69° ± 9.30, in cluster 2 the average VF increase equals 4.28° ± 4.62. Cluster 1 contains only patients (n = 8) who suffered from hemorrhage, cluster 2) consist only of patients (n = 9) in whom VF loss had been caused by infarction.

When using change of detection rate in static perimetry as a treatment outcome variable, cluster analysis resulted in three clusters. The average treatment effect in those clusters equals as follows. Cluster 1: 19.625 % ± 3.64, cluster 2: 28.08 % ± 10.17, cluster 3: 6.30 %. Cluster 2 demonstrating the maximum treatment effect contains only patients (n = 6) in whom the VF defects was caused by infarction. Cluster 1 with the second highest treatment efficacy consists only of patients (n = 4) with lesions due to hemorrhage. Cluster 3 with the lowest gain contains four patients with infarctions and one with hemorrhage. In contrast to clusters 1 and 2 in which all patients performed the treatment with both eyes open, cluster 3 contains only patients who performed the treatment monocularly.

When comparing both outcome measures of change of VFsize in a) degrees of visual angle and b) percentage of detection rate, results are controversial. In kinetic perimetry patients suffering from hemorrhages (cluster 1 kin) demonstrate the highest gain, whereas in static perimetry patients with infarctions (cluster 2 stat) profit the most from treatment.

Unfortunately the influence of the lesion type and ocularity of treatment variables cannot be separated, since in kinetic perimetry both clusters contain some patients of any type of treatment ocularity.

### Transfer of treatment effects to other visual functions than VF change and to performance of activities of daily living

#### Visual acuity

Out of the 20 patients, in 13 (# 1–7, 10, 11, 13, 15, 19, 20) central visual acuity of both eyes had been reduced due to CVA, in seven acuity had remained unchanged. Out of those 13 patients, spontaneous recovery of acuity had occurred before VF treatment in eight (# 1, 3, 5, 6, 7, 13, 15, 20), in two patients (#2, 10) acuity even had worsened some time after CVA, and in three patients no report was given. After VF treatment, partial or complete restoration of visual acuity was measured in ten (# 1–7, 11, 19, 20) of those 13 patients who had suffered from acuity reduction due to CVA, whereas in three of them (# 10, 13, 15) no change was found.

#### Form and color vision

Impairment of central visual acuity due to CVA (in 13 patients) was always associated with a reduction of form (shape) perception in the the fovea and within any possible residual VF of the affected hemifield (s). In three patients (# 14, 16, 18) form recognition was reduced after CVA without a decrease of foveal acuity.

In eight patients CVA had resulted in impairment (# 5, 6, 16, 18–20) or complete loss (#7, 15) of color vision, either foveally or within the affected hemifield(s). Loss of acuity, form or color perception in the affected residual – not completely anopic – VF, caused by cerebral defects, is termed "amblyopia". In only two (# 16, 18) of the eight color amblyopic patients, color amblyopia was not associated with a reduction of central visual acuity, but occurred together with form amblyopia.

After VF treatment in ten patients (# 1–3, 7, 10, 14, 16, 18–20) form perception was improved moderately by an average of 16% [range 10 to 28] as demonstrated by either increasing the detection rate to presentation of oriented lines or/and enhancing the area size within the amblyopic VF where different forms could be discriminated from each other. This improvement of form perception was observed though no specific form treatment had been performed by that time. Out of eight patients suffering from color amblyopia due to CVA, in five cases (#7,16, 18–20) a moderate increase [average 15%; range 7 to 23%] of color perception ability had resulted, too, as the consequence of VF treatment, without any color treatment. The changes were observed when comparing the patient's performance on either the Farnsworth- Munsell 100-hue discrimination test or the size of the color amblyopic VF to color stimuli of equal size, form and luminance before and after VF treatment. In each of those five patients, the ability to discriminate forms had increased, too, as a consequence of pure VF treatment, indicating some kind of stimulus "generalization" effect.

#### Activities of daily living

The ability to perform visual related activities of daily living (ADL) such as reading, avoiding obstacles, orienting in space, walking, riding a bike, manipulating with things in the house or garden and working was evaluated by semi-structured pre- and post-treatment interviews and by the patient's spontaneous communications during treatment, whenever distinct improvements were made. Patients were asked to answer whether their performance had improved or worsened or was unchanged after treatment. Fourteen patients reported improvement of at least five out of the above seven activities, two did not perceive improvements (#8, 15); and four (#2, 11, 13, 17) were not sure. Patients who reported not to have noticed improvements, actually did not demonstrate any improvement when investigating their acuity, form or color discrimination ability. Three out of the four patients not being sure about ADL improvements (#11, 13, 17) did not suffer from acuity, form and color deficits after CVA and did not show any changes after VF treatment. Obviously an increase of those functions is more likely to be detected by the patients than gradual VF enlargements or their own behavioral changes: all but one (#9) patient demonstrated improvement of at least one out three functions tested (visual acuity, form or color perception).

## Discussion

The aim of the present investigation was to introduce a new and efficient automated treatment device and technique to restore VFs after cerebrovascular accidents not suffering from supplementary attention deficits (neglect). In 17 of 20 patients with homonymous hemianopia due to cortical lesions, an average of two sessions per week during a period of little more than eight months of ambulatory treatment with the LRP were sufficient to enlarge VFs by an average of 11.3° ± 8.1 and enhance the detection rate within the defective VF area by 18.6% ± 13.5. Out of twelve patients who had been subjected to both static and kinetic perimetry, in ten stato-kinetic dissociation occurred as a result of treatment, mostly (n = 7) in favor of demonstrating better results in static perimetry outcome. In addition, acuity, form and color perception increased moderately in the majority of the patients who had suffered from a reduction of these functions besides hemianopia due to CVA. Generalization of improvement occurred, though no specific treatment besides superthreshold monochromatic stimulation with small circular LEDs had been performed. Fourteen out of 20 patients reported transfer of treatment effects to improvements of visually guided ADL. These effects were clearly distinguished from spontaneous recovery since the average interval between the lesion and the beginning of therapy was 24.2 months. Only in three patients the interval had been shorter than six months. By comparing size and detection rate in treated and untreated VF areas it was demonstrated that in all cases visual recovery was limited to those parts of the defective VF which had been subjected to visual stimulation. This is in accordance with our early observations when a manual threshold stimulation technique had been used [13-15] for treatment.

### Comparison with earlier studies

The success of the current study, using suprathreshold stimulation in a SRT paradigm to enlarge VFs in hemianopic patients, confirms in part the outcome of earlier studies, using repetitive threshold stimulation or saccadic localization techniques [2,3]. Zihl and von Cramon [2] demonstrated an average VF increase of 10.2° in twelve patients with lesions of the post-geniculate visual pathways obtained within 9 – 37 sessions of repetitively measuring contrast thresholds in an area close to the anopic part of the VF. When using saccadic treatment procedures Zihl and von Cramon [3] in 21 of 55 patients demonstrated VF increases between 6 and 48° which, however, were never observed along the whole anopic field border (as in our patients) but were instead restricted to particular regions. In a replication study by Balliet et al. [4] with twelve patients suffering from hemianopia or quadrantanopia, an average of 36 sessions of threshold or saccadic training failed to reproduce the treatment effects seen by Zihl and von Cramon. They had, however, used much smaller stimuli (6' in diameter instead of 69') than in the original investigations. Confirmation of the treatability of VF defects in patients with cerebral insults, however, was derived from studies using a PC monitor for stimulation [5,16]. Eighty to 300 training sessions resulted in an increase of the stimulus detection rate by 41.6% in nine of eleven patients [16]. The authors, however, made no statements on the corresponding changes of actual VF size. Within a recent study [5] 150 sessions each in 19 patients with post-chiasmatic lesions resulted in an average increase of the detection rate of 19.6%. The VF size after treatment as obtained by conventional static computer perimetry, however, showed an increase of only 0.43°. Though the data of both computer treatment studies [5,16] support the hypothesis that visual perception in hemianopic patients may be improved by treatment, their method of calculating improvements of detection rates has to be regarded with caution. There are two reasons: 1) their data refer to a relative small segment of the total VF which is covered by the monitor (30° × 25°), and 2) their data are calculated as "change over baseline" in which baseline values (before treatment) were taken as 100%. Due to this definition, in a patient who for example had detected two stimuli within the monitor area before treatment, a post-treatment detection of four stimuli would result in an increase of 100%. In contrast, according to our method of calculation a detection rate or improvement of 100% equals to the complete hemifield. Based on this calculation method we found an increase of detection rate (binocular average) due to treatment by 18.6% and a kinetic enlargement by 11.3° (binocular average). When averaging the normal temporal half-field of one eye and the ipsilateral half-field of the other eye, the normal average VF eccentricity equals to 68.5°. An increase of 11.3° by treatment thus corresponds to an average VF increase by 16.4%. Thus, the static and kinetic improvements found in the current investigation are similar, whereas those of Kasten et al. [5] differ considerably from each other: in patients with post-chiasmatic lesions their increase of detection rate equals to 19.6%, contrasted by a change of VF border position of only 0.43°.

In the most recent study [17] using software and a PC monitor for stimulation, 17 hemianopic patients performed visual restoration training during a six-month period. To evaluate the treatment effect, the size of the VF defect before and after treatment was quantified with the help of a scanning laser ophthalmoscope. In none of the patients, however, an explicit homonymous change of the absolute field defect border was observed.

In addition to studies aiming at recovery of VFs in patients suffering from cerebral lesions, few studies were dedicated to the training of compensatory techniques in those patients. Besides the aspired improvements of visual search efficacy, as a side-effect Kerkhoff et al. [18] reported, that after saccadic and visual exploration training, one third of their patients demonstrated an increase in VF size of 5 – 7°. Reading training had a similar effect: In 34% of hemianopic patients who participated in reading training, an average VF enlargement of 5.4° was obtained after 15–24 training sessions, besides enhancements of reading speed and accuracy [19]. In contrast, Pommerenke and Markowitsch [20] did not find any significant VF enlargement after saccadic localization training.

### Stato-kinetic dissociation

When measuring VF in hemianopic patients, different methods may result in different size and shape. This effect is known as stato-kinetic dissociation or "Riddoch phenomenon" [21] and is regarded as a type of amblyopia and – during spontaneous recovery – as a positive prognostic indicator of ongoing improvement, and moreover was regarded to have topodiagnostic value, of occipital lesions, in times before CT or MRI imaging [21] Usually moving stimuli are detected more easily and kinetic perimery results in large VFs. On the other hand, too slow movements (< 3–4°/s) of test stimuli may not be detected [22]. In rare cases the inverse effect of stato-kinetic dissociation was observed: stationary stimuli were detected more easily than moving [23,24]. This inverse effect in some cases is related to lesions in the occipito-temporal region of patients, an area known as V4 which from studies in primates [i.e. [25]] is regarded as sensitive for "motion perception".

In all our patients pre- and post-treatment kinetic perimetry was performed by the same very experienced investigator (author FS) at appropriate stimulus velocities of 3–4° allowing for prompt reaction. Before treatment no stato-kinetic dissociation was observed after the end of spontaneous recovery (eleven of twelve patients in whom both types had been performed). Within the other patient (# 3 with a PTI < 6 months) who was investigated since one month after CVA, kinetic and static VFs also did not differ from each other during spontaneous recovery.

Differences between kinetic and static perimetry occurred only as a consequence of treatment. In only three (patient # 6, 8, 10; cluster C) of them increases in kinetic perimetry were larger than in static (corresponding to the typical Riddoch phenomenon), in seven patients (cluster B) treatment-induced changes were more pronounced in static perimetry. In two of the patients of cluster C (with posterior cerbral artery lesions) actually only kinetic perimetry did show improvements, whereas static perimetry did show a slight VF size reduction instead. The only patient (# 6) of cluster C suffering from a hemorrhage of the middle cerbral artery, however, regained 30° of her kinetic VF, but only 18.5% of her VF measured by static perimetry. Restoration of movement perception is believed to eventually involve phylogenetic older and extrastriate pathways and may occur even if only small neuron populations did survive the insult ([1] and see discussion below). During spontaneous recovery in hemianopia, many authors report a certain sequence: recovery of 1. movement perception, 2. perception of white light, and 3. color perception.

The largest cluster B – showing the inverse Riddoch phenomenon – mainly contains patients suffering from infarctions of the posterior cerebral artery. The higher degree of restoration in detecting static stimuli in this majority of patients may as well result from the special LRP treatment technique which uses only static stimuli. Differences in the amount and type of restoration in individual patients, may in general reflect a variability of activation of cortical and eventually subcortical areas and has to be the subject of further investigations (see discussion below).

### Potential structures and mechanisms involved in recovery

All lesions of our patients were of cortical origin. If cortical structures were involved in the recovery process, too, monocular treatment should also result in VF improvement of the eye occluded during the treatment, since the majority of visual cortex neurons can be activated by stimulation of both eyes. In fact, in six of seven patients who had received monocular LRP treatment, the VF size increased to similar degrees for both, the treated and occluded eyes. In addition our data demonstrate that treatment is more effective under binocular viewing conditions. When treatment was performed with both eyes open the change of detection rate was three to four times higher (clusters 2: 28.08% and 1: 19.625%) than under monocular treatment conditions (cluster 3: 6.30%). Most of visual cortex neurons respond stronger when stimulated binocularly. This supports the hypothesis that binocular cortical areas are engaged in the process of restoration, if they were not the only structures responsible for recovery. A similar observation has been made by Zihl and von Cramon [2] who found improvements of the same magnitude for the treated and the occluded eye. The recovery of visual cortex neurons is the most likely explanation, too, of pronounced improvements of cortical VEPs observed by some authors [26,27] in hemianopic patients after treatment. Further support of the visual cortex being the potential location of recovery, is also given by early animal findings of Cowey [7]. In contrast to a training-induced decrease of the scotoma size after a partial lesion of the visual cortex in monkeys [6,8], no recovery with practice was found when the lesion was located in the retina [7]. Restoration of function, however, strongly depends upon preservation of a certain amount of cortical tissue [28]. Recently Tegenthoff et al. [29] demonstrated the efficacy of their PC-based visual stimulation therapy in partially rehabilitating visual functions in patients with post-traumatic cortical blindness. These patients are generally believed to suffer from permanent blindness [30]. The authors interpreted their positive therapy outcome as a sign of a high degree of neuronal plasticity of the visual cortex. Axonal sprouting over a period of several months [31] and functional reorganization of existing cortical synaptic connections, as seen within the somatosensory system, are believed to be potential candidates for the physiological repair mechanisms of visual recovery after repetitive light stimulation. Eysel and co-workers [32,33] demonstrated that: 1. focal lesions in cat visual cortex induced a short-latency spontaneous enlargement of receptive fields at the border of the lesion and a shift of retinotopy from the region lost by the lesion to surviving cells adjacent to it, and 2. approximately one hour of visual training of those neurons may result in a temporary expansion of the receptive field towards the stimulated side of the receptive field. As a possible mechanism underlying the training effect, the authors suggest that formerly sub-threshold geniculo-cortical synapses became suprathreshold due to a mechanism such as long term potentiation LTP.

In accordance with those findings it is possible that reactivation of surviving neurons within that part of the damaged visual cortex itself (representing the transition zone from intact vision to anopia) is the mechanism underlying treatment-induced recovery phenomena in hemianopic patients [5]. In many of our patients of an earlier investigation and as is i.e. demonstrated in patient # 20 (fig. 6), VF recovery was not restricted to the extent of the transition zone with a gradual decrease of contrast [34]. The final VF gain due to treatment was a multiple of the size of the initial transition zone. This may indicate that numerous initially "silent" neurons outside the transition zone must have been activated by repetitive light stimulation. In addition to primary visual cortex, other structures may be involved in the process of visual restoration, as is demonstrated by a recent report on spontaneous recovery in a patient suffering from bilateral occipital lobe damage. Despite MRI and PET scans still indicated cortical hypermetabolism, the patient's VF in one hemisphere had recoverd [35]. An increase of regional cerebral blood flow rCBF, however, was seen in the pulvinar thalami and the lateral geniculate nucleus [36]. As has been discussed before by several authors in the context of "blindsight", these structures belonging to the extra-striate visual system, may contribute to visual restoration. Further information with respect to the role of subcortical nuclei is expected from our ongoing investigations with the help of functional imaging including fMRI.

### The importance of attention in recovery

In patients with severe damage to visual cortical areas V1 and V2 resulting in a very small remaining VF, fMRI measurements during visual stimulation revealed diminished activation in the striate cortex but increased activation in extrastriate areas of the frontal eye fields, supplementary eye fields and superior parietal cortex, never seen in control subjects [37]. These structures are believed to be part of a network that is related to eye movements as well as spatial attention during visual perception. Independent of the side of residual V1 activation, significant activation of frontal cortical areas was found only within the right hemisphere. When visual functions had recovered completely, fMRI data reveal that activation of the primary visual cortex was re-enhanced (comparable to improvement of cortical VEPs) whereas extrastriate activation had disappeared. These data are in accordance with our hypothesis that the lesioned cortex itself is one of the important locations where recovery takes place and in addition points towards the role of structures related to attention.

The importance of attention for visual recovery during visual stimulation treatment has been stressed by other authors before [2,5,29]. Focusing attention and stimulation on the intact VF or focusing attention on the anopic VF without visual stimulation [38] do not have an effect on VF size. The same is true for visual stimulation during every day life. In 1917 Poppelreuter [22] already commented, that after the initial phase of spontaneous recovery, further improvement can only be acquired by systematic treatment. Successful treatment has to combine attentional and stimulus-related aspects. In the present study in which the majority of patients did profit from therapy, special efforts were undertaken to prevent fatigue and to keep global attention at a high level only when necessary. Spatial attention was selectively attracted to the stimulated VF areas by successively displacing stimulus positions to adjacent LEDs so that the very next stimulus location could be anticipated.

### Transfer of treatment effects on activities of daily living

The aim of enlarging hemianopic VFs by treatment is to improve the patient's daily performance in manipulating things, visually orienting, walking around, driving or reading. In only two studies so far the effects of visual treatment on daily living have been investigated. Within the study of Zihl and von Cramon [3] as in ours, only some of the patients noticed their treatment induced VF enlargement. Unlike in our study, these reports depended on the eccentricity of the VF border. In the study of Kasten et al. [5], out of 30 patients with post-chiasmatic and optic nerve lesions, treated in front of a computer monitor and responding to a questionnaire eighteen (60%) had experienced subjective improvement of vision due to treatment, a percentage close to the 70% in the present study using a reaction perimeter for treatment. Since treatment-induced restoration of VF is attended by improvement of a variety of functions such as visual acuity, incremental threshold and to some extent of form and color vision (of which the change of the latter two functions point to a certain degreee of generalization) VF increase in this study is interpreted as a result of sensory perceptual improvements rather than of changes of detection and response criteria by patients.

In conclusion, visual field treatment in hemianopic patients, using a specially-designed automated-treatment perimeter, resulted in distinct and sustained recovery of part of the visual field in the majority of patients. This treatment was most effective when performed under binocular viewing conditions. Most of the patients reported improvements of visual acuity, form perception and a successful transfer of visual field increase to amelerioration of visually guided activities of daily living. The damaged cortex itself appears to be the most likely structure of visual field recovery. This may, however, require facilitatory influence from structures of the attentional network. Compared to other methods, such as repetitive threshold measurements, saccadic localization or PC training, the treatment performed with the Lubeck Reaction Perimeter provides an efficient, automated technique to obtain a stable partial recovery of visual field areas, which results in better visual performance in everyday life of brain-lesioned patients.

## Competing interests

Fritz Schmielau is holding a United States patent (1996; # 5,534,953) "Training device for the therapy of patients having perception defects" and a European Patent (1997; # EP 0689822) "Training device for treating patients suffering from perception disorders."

## Authors' contributions

FS conceived the study, carried out the assessment and most of the treatment sessions and performed the data evaluation. EKW participated in the design of the study and discussion of the data and helped to draft the manuscript. Both authors read and approved the final manuscript.

## References

[B1] Kölmel HW (1988). Die homonymen Hemianopsien.

[B2] Zihl J, von Cramon D (1979). Restitution of visual function in patients with cerebral blindness. J Neurol Neurosurg Psychiat.

[B3] Zihl J, von Cramon D (1985). Visual field recovery from scotoma in patients with postgeniculate damage. Brain.

[B4] Balliet R, Blood KMT, Bach-y-Rita P (1985). Visual field rehabilitation in the cortically blind?. J Neurol Neurosurg Psychiat.

[B5] Kasten E, Wüst S, Behrens-Baumann W, Sabel BA (1998). Computer-based training for the treatment of partial blindness. Nature Medicine.

[B6] Cowey A, Weiskrantz L (1963). A perimetric study of visual field defects in monkeys. Q J Exp Psychol.

[B7] Cowey A (1967). Perimetric study of field defects in monkeys after cortical and retinal ablations. Q J Exp Psychol.

[B8] Mohler CW, Wurtz RH (1977). Role of striate cortex and superior colliculus in visual guidance of saccadic eye movements in monkeys. J Neurophysiol.

[B9] Weiskrantz L, Cowey A, Passingham C (1977). Spatial responses to brief stimuli by monkeys with striate cortex ablations. Brain.

[B10] Schmielau F (1996). Training device for the therapy of patients having perception defects.

[B11] Schmielau F (1997). Training device for treating patients suffering from perception disorders.

[B12] Wall M, Kutzko KE, Chauhan BC (2002). The relationship of visual threshold and reaction time to visual field eccentricity with conventional automated perimetry. Vision Res.

[B13] Schmielau F, Potthoff RD, Frey D (1990). Erholung von Sehfunktionen als Folge spezifischer Rehabilitationsverfahren bei hirngeschädigten Patienten [abstract]. Bericht über den 37 Kongress der Deutschen Gesellschaft für Psychologie: 23–27 September; Kiel.

[B14] Potthoff RD, Schmielau F (1989). Specific training improves perception in cerebral blindness [abstract]. Perception.

[B15] Potthoff RD, Schmielau F, Steinbüchel N, Pöppel E, von Cramon D, Palitzsch M (1989). Recovery of form and color perception in brain damaged patients requires time and specific training procedures [abstract]. International Symposium 'Brain damage and rehabilitation – a neuropsychological approach' 9–11 October 1989; München.

[B16] Kasten E, Sabel BA (1995). Visual field enlargement after computer training in brain damaged patients with homonymous deficits: an open pilot trial. Rest Neurol Neurosci.

[B17] Reinhard J, Schreiber A, Schiefer U, Kasten E, Sabel BA, Kenkel S, Vontheim R, Trauzettel-Klosinski S (2005). Does visual restitution training change absolute homonymous visual field defects? A fundus controlled study. Br J Ophthalmol.

[B18] Kerkhoff G, Münßinger U, Meier EK (1994). Neurovisual rehabilitation in cerebral blindness. Arch Neurol.

[B19] Kerkhoff G, Münßinger U, Eberle-Strauss G, Stögerer E (1992). Rehabilitation of homonymous scotomata in patients with postgeniculate damage of the visual system: saccadic compensation training. Rest Neurol Neurosci.

[B20] Pommerenke K, Markowitsch HJ (1989). Rehabilitation training of homonymous visual field defects in patients with postgeniculate damage of the visual system. Restor Neurol Neurosci.

[B21] Riddoch G (1917). Dissociation of visual perception due to cortical injuries, with especial reference to appreciation of movement. Brain.

[B22] Poppelreuter W (1917). Die psychischen Schädigungen durch Kopfschuss im Kriege 1914/16 mit besonderer Berücksichtigung der pathopsychologischen, pädagogischen und sozialen Beziehungen Bd 1: Die Störungen der niederen und höheren Sehleistungen durch Verletzungen des Okzipitalhirns.

[B23] Pötzl O, Redlich E (1911). Demonstration eines Falles von bilateraler Affektion beider Occipitallappen. Wien Klin Wochenschr.

[B24] Goldstein K, Gelb A (1918). Psychologische Analysen hirnpathologischer Fälle auf Grund von Untersuchungen Hirnverletzter. I. Abhandlung. Zur Psychologie des optischen Wahrnehmungs- und Erkennungsvorgangs. Z Ges Neurol Psychiat.

[B25] Zeki SM (1974). Functional organisation of a visual area in the posterior bank of the superior temporal sulcus of the rhesus monkey. J Physiol.

[B26] Schmielau F (1989). Kompensation retinaler Defekte beim Binokularsehen: Ein Beispiel neuropsychologischer Forschung in der Medizinischen Psychologie. Focus MHL.

[B27] Sarno S, Erasmus LP, Schlaegel W (2001). Elektrophysiologische Korrelate eines Behandlungserfolgs bei Homonymer Hemianopsie. Neurol Rehabil.

[B28] Lashley KS (1938). Factors limiting recovery after central nervous lesions. J Nerv Ment Disease.

[B29] Tegenthoff M, Widdig W, Rommel O, Malin JP (1998). Visuelle Stimulationstherapie in der Rehabilitation der posttraumatischen kortikalen Blindheit. Neurol Rehabil.

[B30] Bergman PS (1957). Cerebral Blindness. Arch Neurol Psychiat.

[B31] Darian-Smith C, Gilbert CD (1994). Axonal sprouting accompanies functional reorganization in adult cat striate cortex. Nature.

[B32] Eysel UT, Freund HJ, Sabel BA, Winne OW (1997). Perilesional cortical dysfunction and reorganization. Brain Plasticity Advances in Neurology.

[B33] Schweigart G, Eysel UT (2002). Activity-dependent receptive field changes in the surround of adult cat visual cortex lesions. Eur J Neurosci.

[B34] Schmielau F (1996). Partielle Restitution visueller Funktionen durch spezifische Trainingsverfahren bei Patienten mit zerebralen Schädigungen [abstract]. Der Ophthalmologe.

[B35] Ptito M, Dalby M, Gjedde A (1999). Visual field recovery in a patient with bilateral occipital lobe damage. Acta Neurol Scand.

[B36] Ptito M, Johannsen P, Danielsen E, Faubert J, Dalby M, Gjedde A (1997). Alternative neural pathway through pulvinar subserving residual vision following unilateral damage to V1. NeuroImage.

[B37] Rausch M, Widdig W, Eysel UT, Penner IK, Tegenthoff M (2000). Enhanced responsiveness of human extravisual areas to photic stimulation in patients with severely reduced vision. Exp Brain Res.

[B38] Zihl J (1981). Recovery of visual functions in patients with cerebral blindness: effect of specific practice with saccadic localization. Exp Brain Res.

